# Does erotic stimulus presentation design affect brain activation patterns? Event-related vs. blocked fMRI designs

**DOI:** 10.1186/1744-9081-4-30

**Published:** 2008-07-22

**Authors:** Mira Bühler, Sabine Vollstädt-Klein, Jane Klemen, Michael N Smolka

**Affiliations:** 1 Behavioural & Clinical Neuroscience Institute, Department of Experimental Psychology, University of Cambridge, Downing Site, Cambridge, CB2 3EB, UK; 2 Central Institute of Mental Health, Department of Addictive Behavior and Addiction Medicine, J5, 68159, Mannheim, Germany; 3 NeuroImage Nord, Department of Systems Neuroscience, University Medical Centre Hamburg Eppendorf, Martinistr, 52, 20246, Hamburg, Germany; 4 Section of Systems Neuroscience, Department of Psychiatry and Psychotherapy, Faculty of Medicine Carl Gustav Carus, Technische Universität Dresden, Germany

## Abstract

**Background:**

Existing brain imaging studies, investigating sexual arousal via the presentation of erotic pictures or film excerpts, have mainly used blocked designs with long stimulus presentation times.

**Methods:**

To clarify how experimental functional magnetic resonance imaging (fMRI) design affects stimulus-induced brain activity, we compared brief event-related presentation of erotic vs. neutral stimuli with blocked presentation in 10 male volunteers.

**Results:**

Brain activation differed depending on design type in only 10% of the voxels showing task related brain activity. Differences between blocked and event-related stimulus presentation were found in occipitotemporal and temporal regions (Brodmann Area (BA) 19, 37, 48), parietal areas (BA 7, 40) and areas in the frontal lobe (BA 6, 44).

**Conclusion:**

Our results suggest that event-related designs might be a potential alternative when the core interest is the detection of networks associated with immediate processing of erotic stimuli.

Additionally, blocked, compared to event-related, stimulus presentation allows the emergence and detection of non-specific secondary processes, such as sustained attention, motor imagery and inhibition of sexual arousal.

## Background

Redoute proposed a model of sexual behavior, which differentiates four different components: a cognitive, an emotional, a motivational, and a physiological one [1]. The cognitive component comprises an evaluation process during which the incentive value of a stimulus is appraised and attention is directed to it. The physiological component is related to autonomic and endocrinological changes, preparing the individual for sexual behavior in a physiological sense. The emotional component is seen as the subjective experience of hedonic feelings associated with sexual arousal, whereas the motivational component includes the desire to satisfy sexual arousal, resulting in goal-directed sexual behavior.

Over the last years, a growing number of studies have investigated brain processing of visual sexual stimuli with functional magnetic resonance imaging (fMRI) or Positron Emission Tomography (PET). These studies have found that erotic stimuli elicit activity in a widespread neural network, whose different parts have been associated with different components of Redoute's sexual behavior model [1,2]. Cortical structures repeatedly found activated include parietal, occipitotemporal and frontal areas as well as the cerebellum, the insular cortex and the cingulate gyrus. Subcortical areas reported are the basal ganglia, the amygdala, the thalamus and hypothalamus (for an overview see Table 1). High spatial resolution images acquired with 7 T fMRI even allow a clear functional distinction between adjacent subcortical structures such as the anterior caudate and mediodorsal thalamus [3].

**Table 1:** Potential substrates of erotic stimulus processing

				**FA**	**PAR**	**OTC**	**INS**	**CIN**	**CER**	**BAG**	**AMY**	**HYP**	**THA**
***fMRI studies***	***contr***	***stim***	***des***										

**Arnow et al. 2002**	spo	vid	block	√**		√**	√**	√**	√	√**		√**	
**Beauregard et al. 2001**	ntr	vid	block		√	√		√	√		√	√	
**Ferretti et al. 2005**	spo	img	event (3s)	√	√	√	√	√			√		
	spo	vid	block	√	√	√	√	√			√	√	√
**Gizewski et al. 2006**	ntr	vid	block	√		√	√	√	√		√	√	√
**Hamann et al. 2004**	ntr	img	block	√	√	√		√	√*	√	√*	√*	
**Karama et al. 2002**	ntr	vid	block	√		√	√	√		√	√	√*	√*
**Kim et al. 2006**	ntr	vid	block	√		√	√	√		√			
**Mouras et al. 2003**	ntr	img	block	√	√	√		√	√				
**Park et al. 2001**	ntr	vid	block	√		√	√	√		√			√
**Stark et al. 2005**	ntr	img	block			√				√	√	√	√
**Walter et al. 2008**	ntr, emo	img	event (5s)	√	√	√		√		√	√	√	

***PET studies***													

**Redoute et al. 2000**	*ntr hum*	*img & vid*	*block*	√	√	√	√	√	√	√		√	√
**Stoleru et al. 1999**	*ntr*	*vid*	*block*	√		√	√	√		√			

FA: Frontal areas; PAR: Parietal regions; OTC: Occipitotemporal cortex; INS: Insular cortex; CIN: Cingulate gyrus; CER: Cerebellum; BAG: Basal ganglia; AMY: Amygdala; HYP: Hypothalamus; THA: Thalamus; contr: control condition; stim: stimulus material; des: fMRI design; hum: humorous; ntr: neutral; spo: sports; emo: emotional pictures; vid: video; img: still images; * significant activation observed only in males; ** penile turgidity-correlated activations

Differences in stimulus induced brain activity reported by these studies might be related to stimulus content (sexual intensity: slightly erotic stimuli vs. pornographic material), presentation mode (static images vs. film sequences) and design type (presentation length of the stimulus material: blocked design vs. event-related design). The differences arising due to stimulus content and presentation mode have already been addressed by two imaging studies, one using videos and static stimuli of graded intensities [1], the other comparing activation elicited by video clips that lead to sexual arousal and penile erection compared to static images that induced sexual arousal without erection [4]. Whether emotion-inducing stimuli should be presented in a blocked or event-related fashion during fMRI is still a matter of debate. Especially in emotion research, the presentation duration appears to be important, not only from a methodological point of view, but also in respect of differences in information processing. Methodologically, blocked designs are superior in detecting activated brain areas, while event-related designs are superior in terms of estimation of the hemodynamic response function [5]. Blocked designs are often used due to their easier implementation since randomization, jittering and spacing of different stimulus categories is not necessary. Furthermore, artefacts can be more easily detected by visual inspection of the data time course [6]. Event-related designs are methodologically more demanding but allow for the randomization of stimulus presentation, thereby reducing confounds caused by stimulus order predictability [7,8]. They further allow sorting trials post hoc according to behavioral responses, making this design more flexible than the blocked design [9,10]. In respect of information processing, blocked designs differs from event-related designs. In blocked designs stimulus presentation length is prolonged and the occurrence of consecutive stimuli in a block is fully predictable [8]. Therefore block designs might more readily activate cognitive processes such as sustained attention as well as top-down regulation mechanisms including inhibition of sexual arousal. This could decrease emotional involvement and hence change the underlying brain activation [11].

Despite several excellent publications concerning theoretical methodological fMRI design considerations [5,12-14] only a few attempts have been made to vary the design type while keeping the stimulus material and the experimental task constant. These studies compared blocked and event-related designs using different visual stimuli [15], a finger opposition task [14], auditory stimuli of different frequencies [16] or higher cognitive tasks, such as semantic judgment [17]. Only one study focused on emotional processing and to what extent brain activation elicited by stimuli depicting fearful and disgusting scenes depends upon the presentation design [11]. In both conditions event-related in contrast to blocked stimulus presentation elicited greater activation in the insular and orbitofrontal cortex.

We know from everyday life that a split second is often enough to decide whether a person is attractive or a scene is erotic. Behavioral studies support this idea by showing that even sexual stimuli presented to the subconscious influence later processing [18,19] or can be used as conditioned stimuli in a classical conditioning paradigm [20]. Furthermore, correlates of erotic stimulus processing have been detected as early as 300 ms after stimulus presentation as shown in a recent electroencephalography (EEG) study by van Lankfeld et al. [21].

Whether information processing is altered when erotic images are presented only for a brief period of time has not yet been addressed by existing imaging studies. A review of the literature on visually evoked sexual arousal revealed that fMRI studies have mainly used blocked stimulus presentation. None of these studies implemented an event-related design with brief presentation times of around one second.

In this study, we compared activation patterns elicited in an event-related design when erotic pictures are briefly (750 ms) presented with brain activity elicited by blocked stimulus presentation in which one block lasts around 20 s. We expected to identify an overlapping network for the blocked and the event-related design reflecting the neuroanatomical correlates of all four components of Redoute's [1] neurobehavioral model of sexual arousal. We hypothesized that the blocked design is superior in identifying areas involved in cognitive aspects of erotic stimulus processing, such as sustained attention and inhibition of sexual arousal. Furthermore, we anticipated for the event-related design that activation would be more pronounced in structures associated with the motivational and emotional component of Redoute's [1] neurobehavioral model, due to less influence from modulating cognitive processes.

## Methods

### Participants

10 healthy volunteers participated in the study. To keep the group of participants as homogenous as possible, we included only right-handed, heterosexual men (mean age 32 years, Standard Deviation (SD) 5 years). Handedness was assessed via the Edinburgh Handedness Inventory [22]. Only right-handed participants with a laterality quotient (LQ) greater than +50 were included in the study (mean LQ +82).

All participants provided informed written consent according to the Declaration of Helsinki. The study was approved by the Ethics Committee of the University of Heidelberg. Participants were recruited by public announcement at the Central Institute of Mental Health. Medical, psychiatric and substance abuse histories were controlled for in a short screening interview.

### Imaging study

#### Stimuli

To induce sexual arousal, we used 18 erotic pictures; 18 neutral images were used for comparison purposes. All stimuli were taken from the International Affective Picture System (IAPS) [23]. We included neutral stimuli with the following IAPS catalogue numbers: 2514, 2575, 2880, 5531, 5740, 6150, 7034, 7080, 7100, 7140, 7175, 7190, 7205, 7233, 7235, 7491, 7500 & 7710; erotic stimuli were: 4002, 4180, 4210, 4232, 4250, 4300, 4310, 4607, 4608, 4651, 4652, 4658, 4664, 4670, 4680, 4683, 4800, 4810.

### fMRI design

All participants took part in two fMRI sessions at intervals of at least one week to reduce memory artefacts occurring due to the repeated presentation of the stimuli. The same stimulus set was presented during both fMRI sessions using either a blocked or an event-related design. In both designs stimulus presentation (erotic stimuli + neutral stimuli + fixation) was comparable in duration (event-related design: 9.11 min, blocked design: 7.59 min). The order of sessions was counterbalanced between participants. In both sessions participants were instructed to passively view the images and not to inhibit their emotions.

#### Blocked stimulus presentation

The 36 images were grouped in blocks of three stimuli, resulting in six blocks for each category. All stimuli were displayed for 6.6 s (i.e., the duration of one block was 19.8 s). The 12 blocks were presented in a pseudo-randomized order and separated by a passive fixation condition of 19.8 s duration. Previously a similar design has successfully been used in our lab in studies on cue reactivity [24].

#### Event-related stimulus presentation

The same 36 stimuli used in the blocked design were also presented in an event-related design. Images were arranged in individually randomized orders and presented for 750 ms with randomly jittered intertrial intervals (ITI), which allowed for the sampling of the Blood Oxygen Level Dependent (BOLD) signal at different peri-stimulus time intervals. The length of the ITI was uniformly distributed and ranged from 9.9 s to 19.8 s. A fixation cross was presented during the ITI. Such an fMRI design has previously been successfully used in our group in studies on processing of emotional stimuli [25-27].

### Data acquisition

Scanning was performed with a 1.5 T whole-body tomograph (Magnetom VISION; Siemens, Erlangen, Germany) equipped with a standard quadrature head coil. For fMRI, 24 slices were acquired every 3.3 s (4 mm thickness, 1 mm gap) using an Echo Planar Imaging (EPI)-Sequence (TR = 1.8 ms, TE = 66 ms, α = 90°) with an in-plane resolution of 64 × 64 pixels (Field of View (FOV) 220 mm). FMRI slices were oriented axially parallel to the anterior commissure-posterior commissure (AC-PC) line according to Talairach and Tournoux. A morphological 3D T1-weighted magnetization prepared rapid gradient echo (MPRAGE) image data set (1 × 1 × 1 mm^3 ^voxel size, FOV 256 mm, 162 slices, TR = 11.4 ms, TE = 4.4 ms, α = 12°) covering the whole head was acquired for anatomical reference.

### Data analysis

Data were analyzed with Statistical Parametric Mapping (SPM99, Wellcome Department of Imaging Neuroscience, University College London, London, UK). The first five images were discarded in order to reduce T1 saturation effects. All individual data were spatially realigned to correct for head movement. Slice time correction was performed in order to minimize temporal differences in slice acquisition. The structural T1 data set was then coregistered to the first functional T2* image before being spatially normalized to a standard template (Montreal Neurological Institute (MNI) brain) using a 12-parameter affine transformation with additional non-linear components. The same non-linear transformation was subsequently applied to the functional T2* data and voxels were resampled at a resolution of 3 × 3 × 3 mm. The functional data were smoothed using an isotropic Gaussian kernel for group analysis (12 mm full-width at half-maximum).

First level statistics were performed by modelling both conditions (erotic and neutral stimuli) as explanatory variables within the context of the general linear model on a voxel by voxel basis (blocked design: boxcar function convolved with a hemodynamic-response function; event-related design: delta functions convolved with a synthetic hemodynamic response function plus temporal derivatives). Individual contrast images (erotic vs. neutral images) were computed separately for the blocked and event-related design and were then included in second level analyses.

For both the blocked and event-related design we identified brain regions that showed increased activation during presentation of erotic stimuli compared to neutral stimuli and vice versa using SPM F-contrasts. Task-related brain activity was regarded as significant if the probability of a Type I error was < 0.05 (two-tailed, False Discovery Rate (FDR) corrected) in 10 adjacent voxels. F-scores were transformed to T-scores by extracting the root. To distinguish posthoc between directions of the effect (erotic > neutral or erotic < neutral) we applied T-contrasts.

The analysis of differences in brain activity during event-related and blocked stimulus presentation was restricted to areas showing robust activation related to at least one of both designs. For this purpose a mask was generated using ImCalc implemented in SPM. We included all voxels activated during event-related and/or blocked stimulus presentation using a threshold of *p *< 0.01 (two-tailed, uncorrected) and a cluster size of at least 10 voxels. We applied this liberal threshold when generating the mask image to reduce erroneous exclusion of brain regions related to the task performance (lower probability of Type II errors). Moreover, the resulting increase of the volume of interest (VOI) made the analysis more conservative. Then, activation patterns during event-related and blocked stimulus presentation inside this VOI were compared using paired t-tests. The statistical threshold was set to *p *< .05 (two-tailed, FDR corrected for the VOI) and a cluster size of at least 10 voxels.

To plot the BOLD response (mean ± standard error of the mean (SEM)) during processing of erotic and neutral stimuli in the blocked and event-related design, we extracted the BOLD signal change of single peak voxels from the SPM contrast images (erotic – fixation; neutral – fixation) using an in-house software running under Matlab (The MathWorks, Natick, Massachusetts, USA) (Figure 2).

### Behavioral measures

Following the second fMRI session, all 36 images used during the scanning sessions were shown to the participants outside the scanner. After presenting the stimuli for 6.6 s on a computer monitor, valence and arousal were assessed with the computerized form of the non-verbal pictorial Self-Assessment Manikin (SAM) [28]. Statistical analyses of the behavioral data were conducted with the statistical software package SPSS (version 12.0, SPSS Inc., IL). Normal distribution of the behavioral data was assessed with the Kolmogorov-Smirnov test. Valence and arousal ratings of erotic stimuli were compared with the corresponding ratings of the neutral stimuli using a paired t-test.

## Results

### Behavioral data

All behavioral data were normally distributed (K-S Z < 0.51, p > .190), thus parametric statistics were applied.

The mean levels of reported arousal assessed with the SAM were 6.75 (± 1.71) for the erotic stimuli and 3.80 (± 0.94) for the neutral stimuli, respectively. These differences in arousal were significant (T_9 _= 4.28, p = .002).

The mean ratings in the valence condition were 7.52 (± 0.91) for the erotic stimuli and 4.98 (± 0.34) for the neutral stimuli. Erotic stimuli were rated as significantly more pleasant compared to neutral stimuli (T_9 _= 7.71, p < .000).

### Imaging data

#### Blocked design

##### Erotic stimuli – neutral stimuli

**Figure 1:**
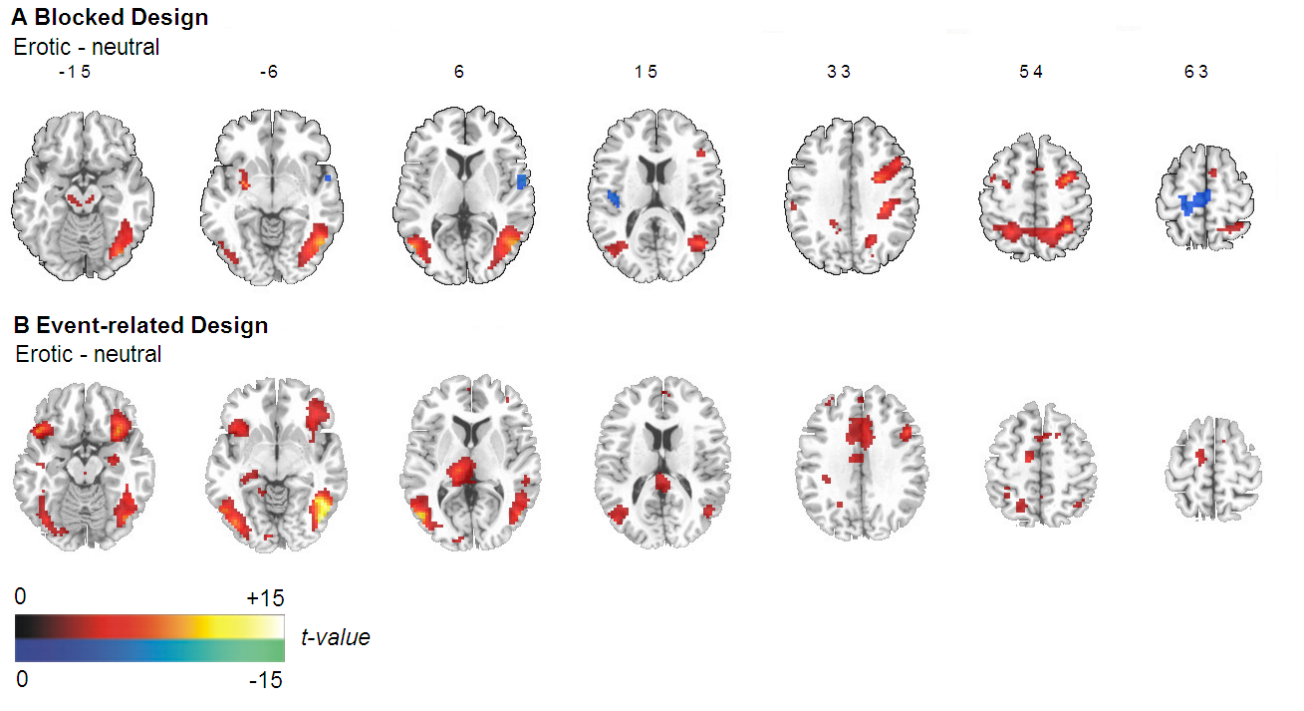
Brain areas activated by erotic compared with neutral stimuli or vice versa in the blocked and in the event-related design (for illustrative purposes *p*_*uncorr*. _< 0.01; two-tailed; |T| > 3.25; cluster size ≥ 10 voxels).

The fMRI BOLD response to erotic compared to neutral stimuli was significantly different (|T| > 4.77, *p*_*FDR *_< .05) in several brain regions. Greater activation in response to erotic stimuli was found in the right (BA 19, 37) and left (BA 37) occipitotemporal cortex, the right (BA 40) and left (BA 7) parietal lobe as well as in both frontal lobes (right: BA 6, 48; left: BA 6). The response to erotic stimuli was also more pronounced in the left cerebellum. In total 2419 voxels showed significantly higher activity during presentation of erotic compared to neutral stimuli (Table 2 and Figure 1A).

**Table 2:** Brain areas activated in the blocked and event-related design in response to erotic compared to neutral images

		**Blocked design**						**Event-related design**					
**Brain area**	**Side**	**Cluster size**	**BA**	**MNI**			**T**_max_	**Cluster Size**	**BA**	**MNI**			**T**_max_
				**x**	**y**	**z**				**x**	**y**	**z**	

**Frontal lobe**	**R**	172	6 48	33 42	-3 15	51 27	9.30 7.12	11	44	48	15	33	6.27
	**L**	48	6	-24	-9	51	7.11	14	6	-9	-15	57	7.54

**Orbitofrontal lobe/Insula**	**R**							116	38 47	36 39	18 42	-15 -3	8.69 5.47
	**L**							81	38 48	-39	18	-12	8.97

**Parietal lobe**	**R**	159	40	36	-48	54	7.67						
	**L**	37	7	-21	-54	54	5.77	16	3	-27	-18	42	6.53

**Temporal lobe/rolandic operculum**	**R**	14	48	63	0	6	-6.18						
	**L**							10	20	-39	-18	-21	5.66

**Occipitotemporal lobe**	**R**	414	37 19	54 42	-63 -75	0 -15	14.68 7.25	379	37 19	48 48	-57 -72	-3 -3	13.77 13.75
	**L**	70	37	-51	-66	3	9.49	266	37 19	-54 -39	-66 -75	3 -9	12.52 8.14

**Thalamus**	**L**							63		-9	-24	6	7.08

**Cingulate gyrus**	**R**							68	24 32	6	12	42	7.37

**Cerebellum**	**L**	48		-3	-72	-27	7.32	63		-9	-24	6	7.08

*p*_*FDR *_< .05; two-tailed; blocked design: |T| > 4.77; event-related design: |T| > 4.69; cluster size ≥ 10 voxels; BA: Brodmann area; MNI: Montreal Neurological Institute; T_max_: Student's T-value (local maximum); L: left; R: right

##### Neutral stimuli – erotic stimuli

Higher activity during presentation of neutral control stimuli compared to erotic images was only found in the right temporal lobe.

#### Event-related design

##### Erotic stimuli – neutral stimuli

The fMRI BOLD response elicited by erotic compared to neutral stimuli was significantly different (|T| > 4.69, *p*_*FDR *_< .05) in a widespread network comparable to that activated in the blocked design. A higher BOLD response to erotic stimuli was found in occipitotemporal (left and right: BA 19, 37) and temporal regions (left and right: BA 20), in both frontal lobes (right: BA 44; left: BA 6) and the left parietal cortex (BA 3). Beside these regions, we found significantly more brain activity during presentation of erotic stimuli in the left cerebellum. Regions only significantly activated during event-related presentation of stimuli included the cingulate gyrus bilaterally (left: BA 23, 24; right: BA 24), the right insula merging with orbitofrontal cortex (BA 38, 47) as well as the left insula (BA 38, 48) and the thalamus of the left hemisphere (Table 2). Overall, 3003 voxels showed significantly higher activity during presentation of erotic compared to neutral stimuli (Table 2 and Figure 1B).

##### Neutral stimuli – erotic stimuli

When comparing the BOLD response elicited by neutral stimuli compared to erotic stimuli in the event-related design none of the brain areas showed significantly higher activity during presentation of neutral stimuli.

#### Blocked vs. event-related design

Task-related brain activity (*p *< .01, two-tailed, uncorrected) during at least one of both design conditions was found in 6308 voxels, i.e., a volume of 170 ml. The following analyses concerning differences between the design types were restricted to this VOI. A direct comparison revealed significantly different BOLD activity (|T| > 3.66, *p*_*FDR *_< .05) during blocked compared to event-related stimulus presentation in 650 voxels, i.e., 10.3% of the total VOI.

**Figure 2:**
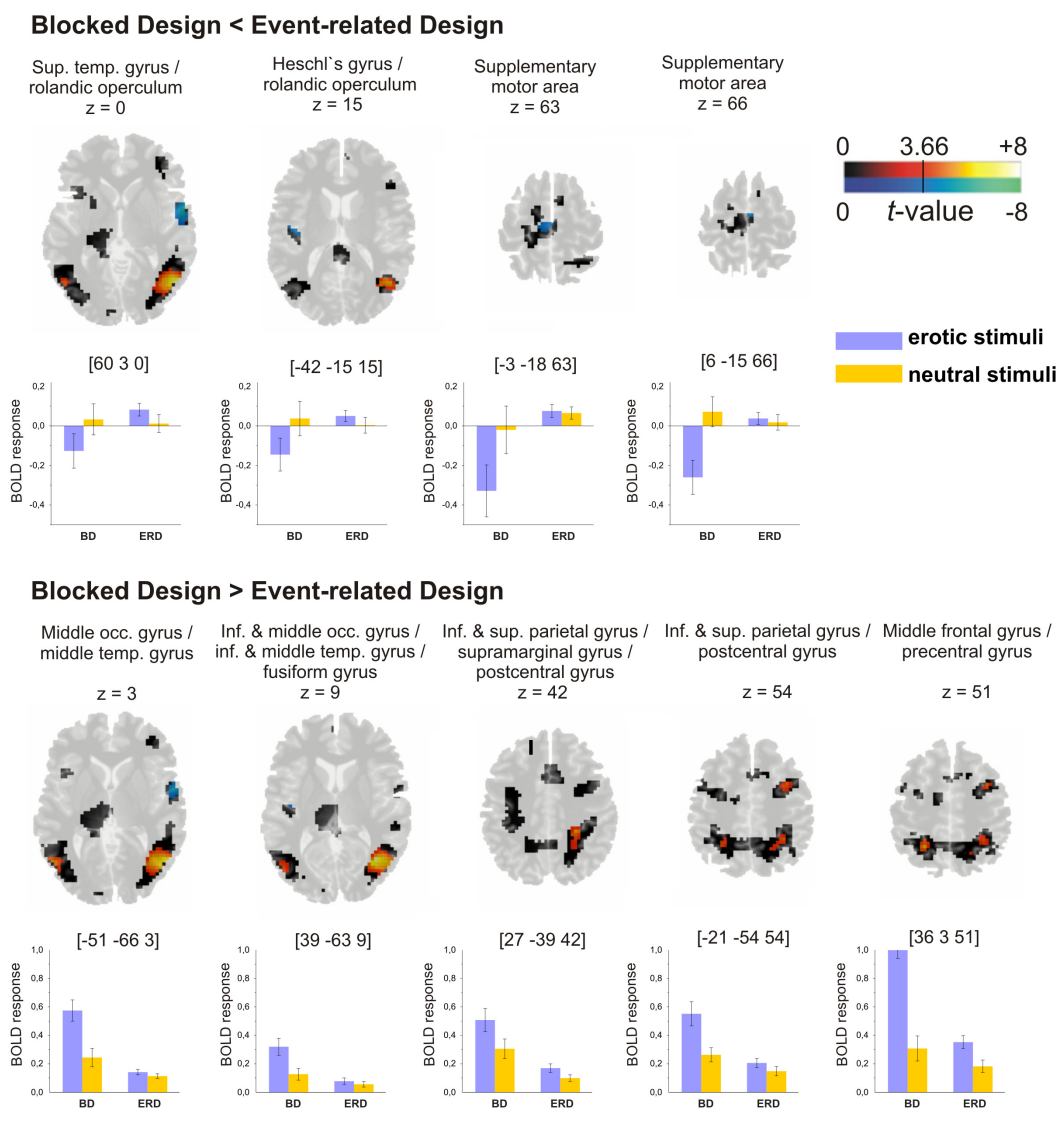
**Activation maps (upper rows) for the comparison between the two design types (*p*_*FDR *_< 0.05 corrected for the volume of the entire mask (two-tailed; |T| > 3.66) and BOLD response data (lower rows) of single peak voxels (mean ± SEM) during processing of erotic and neutral stimuli in the blocked and event-related design. ***BD: Blocked Design; ERD: Event-Related Design*.

Blocked compared to event-related presentation revealed significantly more pronounced BOLD activity in occipitotemporal regions (right: BA 19, 37; left: BA 37), in parietal areas of both hemispheres (right: BA 7, 40; left: BA 7) and the right frontal lobe (premotor areas) (BA 6, 44) (Figure 2; Table 3, left panel).

**Table 3:** Brain areas showing a differential response for the comparison between blocked and event-related presentation of erotic vs. neutral stimuli

**Lobe**	**Side**	**Brain area**	**Cluster size**	**BA**	**MNI**			**T**_max_
					**x**	**y**	**z**	
**Frontal lobe**	**R**	Middle frontal gyrus/Precentral gyrus	18	6 44	36	3	51	4.82
	**L**	Supplem. motor area	13	6	6 -3	-15 -18	66 63	-4.34 -3.94

**Parietal lobe**	**R**	Inf. & sup. parietal gyrus/Supramarginal gyrus/Postcentral gyrus	94	7 40	27	-39	42	5.66
	**L**	Inf. & sup. parietal gyrus/Postcentral gyrus	14	7	-21	-54	54	5.87

**Temporal lobe**	**R**	Sup. temp. gyrus/Rolandic operculum	54	48	60	3	0	-6.02
	**L**	Heschl's gyrus/Rolandic operculum	13	48	-42	-15	15	-4.44

**Occipito- temporal lobe**	**R**	Inf. & middle occ. gyrus/Inf. & middle temp. gyrus/Fusiform gyrus	401	37 19	39	-63	9	8.07
	**L**	Middle occ. gyrus/Middle temp. gyrus	43	37	-51	-66	3	5.51

*p*_*FDR *_< .05; two-tailed; |T| > 3.66; cluster size ≥ 10 voxels

BA: Brodmann area; MNI: Montreal Neurological Institute; T_max_: Student's t-value (local maximum); L: left; R: right; positive T values indicate higher brain activation during blocked design compared with event-related design whereas negative T values indicate higher brain activation during event-related design compared with blocked design

Unlike locked presentation, event-related presentation elicited higher brain activity in the right temporal lobe (BA 48), the left insula/rolandic operculum (BA 48) and bilateral frontal areas (supplementary motor areas) (BA 6) (Figure 2; Table 3, right panel).

## Discussion

To clarify how the experimental design affects stimulus-induced brain activity in response to erotic compared to neutral stimuli in fMRI, we compared the presentation of such stimuli using a brief stimulus duration (0.75 s) in an event-related design with longer presentation durations in a blocked design (19.8 s).

### Blocked and event-related design

In both designs we found increased activation in occipitotemporal regions, more precisely in BA 19/37, in response to erotic stimuli. Increased activation in occipitotemporal regions has been previously reported in response to the presentation of erotic films [29,30]. Additional findings supported the interpretation that increased brain activation in occipitotemporal regions is not specific for erotic stimulus material per se but can be observed in response to emotionally laden stimuli in general. Therefore this could reflect motivated attention [31,32] and, as such, be considered as neural correlate of the cognitive component of Redoute's [1] neurobehavioral model of sexual arousal.

Activation in the inferior frontal gyrus (event-related design: BA 44, Broca's area; blocked design: BA 48) was reported by Beauregard et al [33] during attempted inhibition of sexual arousal. He related the activation to internal verbalization processes supporting the inhibition process. Although, participants were not explicitly instructed to inhibit sexual arousal, they could have tried to do so. Attempted inhibition of sexual arousal is related to appraisal processes and as such may be considered part of the cognitive component of Redoute's [1] model.

We observed a significant increase in the BOLD response in the premotor cortex (BA 6) for both designs. This area is involved in planning and mental rehearsal of a movement [34]. Animal research suggests that premotor cortex activation reflects the degree of motivation, presumably related to the motor readiness [35]. The motivational component comprises processes that direct behaviour to a sexual goal. Thus increased activation in the premotor cortex could be associated with the motivational component of Redoute's [1] model.

### Blocked design

In the blocked, but not in the event-related design, we found a significant increase in activation in the inferior and superior parietal lobes (BA 7, 40). These structures have been shown to play a role in attentional processes [36]. In particular, the right parietal cortex has been shown to be part of an interoceptive attention system that monitors bodily states [37] and lateral regions of the posterior parietal cortex have been linked to sustained states of peripheral attention [38]. These finding suggest that parietal activation could be added to the cognitive component of Redoute's [1] model.

### Event-related design

In accordance with previous findings concerning the processing of visual sexual stimuli using the event-related design we found activation in the insular and somatosensory cortices as well as in the thalamus. These structures have been proposed to play a role in the perception of one's own behavioral responses and representations of bodily states and are associated with the somatosensory processing pathway [4,39]. Furthermore, insula activation has been linked to cross-modal matching and could reflect cross-modal transfer of visual input to imagined tactile stimulation [39]. Neuroimaging data expanded these results by showing that activation in the left insula is correlated with orgasm quality in women [40]. These findings support the role of the insula as an interoceptive monitoring system, which is activated when stimuli are associated with strong somatic experiences. We interpret the activation in these areas as the neural correlates of the emotional component of Redoute's [1] neurobehavioral model of sexual arousal, referring to the specific hedonic quality of sexual arousal and the perception of associated bodily changes.

The anterior cingulate cortex is part of the affective division, which modulates emotional and autonomic responses [2] and has previously been shown to be activated in response to erotic stimuli [29,30]. According to Mouras and colleagues [41], the activated caudal part of the anterior cingulate gyrus (BA 24) could constitute a neural correlate of the motivational component which comprises processes that guide behaviour to a sexual goal. Activation of the more rostral part (BA 32) could be assigned to the autonomic component, which includes various autonomic and endocrine responses, leading the subject to a state of physiological readiness for sexual behaviour. A recent study which aimed to distinguish specific sexual and general emotional effects in fMRI during erotic picture viewing, supported the idea, that activations in the anterior cingulate cortex were modulated by both, sexual intensity and emotional valence [42].

The supplementary motor area (BA 6) has been suggested to be a motor-limbic interface important to the transformation of visually triggered emotional experiences into motor actions including erectile responses [43,44]. The supplementary motor area might be regarded as part of the motivational component of Redoute's [1] model, transforming salient visual inputs into corresponding goal-directed behavior.

### Blocked design – event-related design

The statistical comparison between the blocked and the event-related fMRI design revealed a significantly higher BOLD response during blocked presentation in the inferior and superior parietal lobes. As mentioned in the previous paragraph, these regions play a role in attentional processes [36]. Particularly sustained activity in lateral regions of the posterior parietal cortex has been linked to sustained states of peripheral attention [38]. Innervations from the anterior cingulate to the inferior and superior parietal lobe allow interactions with areas related to motivational processes, helping to maintain attention to stimuli when appraised as motivationally relevant cues [45]. It seems very likely that the blocked design requires sustained attention due to its longer stimulus presentation times.

We found further differences in occipitotemporal areas, which are supposedly not specifically involved in the processing of erotic or sexual material, but have been shown to be related to the processing of emotionally laden stimulus material in general. These regions seem to be involved in motivated attention, prompted by motivationally relevant cues, which direct attention and facilitate the processing of survival-relevant stimuli [31,32].

We observed significantly more activation in the right frontal lobe including premotor areas and the inferior frontal gyrus in the blocked, compared to event-related, design. A neural network including the premotor cortex, as well as superior and inferior parietal areas among others [34,46,47], has not only been shown to be involved in action execution but also in imagined actions. As such, the increased activation could reflect enhanced imagination of erotic acts enabled through increased presentation length in the blocked design. The right inferior frontal gyrus is of uttermost importance in response inhibition. Increased activation in this brain area might reflect the necessity of increased inhibitory control of erectile responses in the blocked design due to longer stimulus presentation times.

### Event-related design – blocked design

Comparing event-related with blocked presentation of erotic vs. neutral stimuli, we found significant differences in the right superior temporal gyrus/rolandic operculum and the left Heschl's gyrus/rolandic operculum. These differences are caused by a substantial negative response in the blocked design (erotic – neutral stimuli) compared to a slight and non-significant positive BOLD response in the event-related design. Our data show that these results are based on a deactivation in response to erotic stimuli (compared to fixation) in the blocked design, rather than on an increase in activation in response to neutral stimuli.

Deactivation of the primary auditory cortex (Heschl's gyrus) differentiates visual imagery from visual perception as shown by Amedi and colleagues [48]. They found that visual imagery elicits isolated activation of visual cortical areas with concurrent deactivation of "irrelevant" or "disturbing" sensory processes, thereby possibly avoiding a disruption of the internal image created by the "mind's eye", e.g., by scanner noise. Although we presented visual stimuli to our subjects, it can be speculated that erotic material could elicit visual imagery beyond the physical image presented during scanning.

Furthermore, significant differences were observed for the supplementary motor area (BA 6), which similarly resulted from a substantial negative response in the blocked design in response to erotic – neutral stimuli. This region has been shown to be involved in the control of movements triggered by visual cues with negative emotional content [43] and the generation of erectile responses to the presentation of sexually stimulating photographs [44]. These preparatory motor-related actions are possibly less problematic in the event-related design, due to short presentation times, but may have to be actively suppressed during blocked stimulus presentation.

In summary, the results of the comparison of a blocked presentation of erotic stimuli vs. neutral stimuli compared to an event-related presentation suggest that the activation differences between the design types result predominantly from changes (either increase or decrease) in the BOLD characteristics in response to visual erotic stimulation in the blocked design.

Increased activation in occipitotemporal, frontal and parietal brain areas in response to erotic stimuli in the blocked design probably reflects sustained visual attention to motivationally relevant stimuli, motor imagery as well as increased response inhibition of motor-related actions such as erection responses.

Decreased activation in temporal and supplementary motor areas in response to erotic stimuli in the blocked design could be related to deactivation of "irrelevant" sensory systems and the successful suppression of motor-related actions such as erection responses [44].

### Limitations

Several methodological limitations need to be addressed. First, to reduce effects of motor-related processes on the BOLD response, we did not include a performance measure that controls for shifts in attention during the fMRI scan. Instead, we compared visual presentation with the resting condition and verified that, during the entire scanning procedure, all subjects showed substantial brain activation in occipital and fronto-parietal regions, indicative of attentional processing of visual information. Second, the sample size of this study is small, thus moderate signal increases might be missed due to a high probability of a Type II error. On the other hand, finding significant BOLD activations in such a small sample may indicate a strong effect size. Third, neutral images similar to those used in most published studies were used as control stimuli. Therefore we cannot differentiate between emotional and sexual components in the neural network underlying sexual arousal [42]. Forth, only male participants were included in the study to minimize the variability in cue-elicited brain activation. This certainly affects the generality of the results, as women have shown to differ from men in their preference of sexual stimuli [49]. Future studies are strongly needed to clarify whether the results of this study hold not only for men but also for women and/or individuals with homosexual orientation. Last, we were not able to detect a significant increase in activation in the amygdala, hypothalamus or striatum in response to erotic images, as previously reported by other studies. This finding could be attributed either to the small sample size and hence lower detection power in our study or the use of static images instead of films.

## Conclusion

Our results show that event-related presentation of erotic stimuli for only several hundred milliseconds activates brain circuits known to be relevant in processing of erotic images.

Blocked, compared to event-related, presentation of erotic stimuli additionally allows for the emerging and detecting of non-specific secondary processes, including sustained attention to relevant stimuli, motor imagery and intentional suppression of motor-related actions such as erection responses. Although sexual arousal and attention are important components of sexual behavior, it is difficult to control for these unspecific processes and they might interfere with the detection of networks associated with immediate processing of erotic stimuli.

In summary, our findings suggest that event-related designs might be a potential alternative when the core interest is the detection of networks associated with immediate processing of erotic stimuli.

## Competing interests

The authors declare that they have no competing interests.

## Authors' contributions

MB has been responsible for designing the study, analysing the data and writing up the manuscript. SVK carried out the acquisition and the analysis of the data and commented on the written drafts of the manuscript. JK commented on the written drafts of the manuscript and participated in scientific discussions about the content of the manuscript. MNS designed and coordinated the study, supervised the data analysis and the writing process. All authors have read and approved the final manuscript.

## References

[B1] Redoute J, Stoleru S, Gregoire MC, Costes N, Cinotti L, Lavenne F, Le Bars D, Forest MG, Pujol JF (2000). Brain processing of visual sexual stimuli in human males. Hum Brain Mapp.

[B2] Stoleru S, Gregoire MC, Gerard D, Decety J, Lafarge E, Cinotti L, Lavenne F, Le Bars D, Vernet-Maury E, Rada H, Collet C, Mazoyer B, Forest MG, Magnin F, Spira A, Comar D (1999). Neuroanatomical correlates of visually evoked sexual arousal in human males. Arch Sex Behav.

[B3] Walter M, Stadler J, Tempelmann C, Speck O, Northoff G (2008). High resolution fMRI of subcortical regions during visual erotic stimulation at 7 T. MAGMA.

[B4] Ferretti A, Caulo M, Del Gratta C, Di Matteo R, Merla A, Montorsi F, Pizzella V, Pompa P, Rigatti P, Rossini PM, Salonia A, Tartaro A, Romani GL (2005). Dynamics of male sexual arousal: distinct components of brain activation revealed by fMRI. Neuroimage.

[B5] Birn RM, Cox RW, Bandettini PA (2002). Detection versus estimation in event-related fMRI: choosing the optimal stimulus timing. Neuroimage.

[B6] Aguirre GK, D'Esposito M, Moonen CTW and Bandettini PA (1999). Experimental design for brain fMRI. Functional MRI.

[B7] Donaldson DI, Buckner RL, Jezzard P, Matthews PM and Smith SM (2001). Effective paradigm design. Functional MRI.

[B8] Zarahn E, Aguirre G, D'Esposito M (1997). A trial-based experimental design for fMRI. Neuroimage.

[B9] Carter CS, Braver TS, Barch DM, Botvinick MM, Noll D, Cohen JD (1998). Anterior cingulate cortex, error detection, and the online monitoring of performance. Science.

[B10] Wagner AD, Schacter DL, Rotte M, Koutstaal W, Maril A, Dale AM, Rosen BR, Buckner RL (1998). Building memories: remembering and forgetting of verbal experiences as predicted by brain activity. Science.

[B11] Schafer A, Schienle A, Vaitl D (2005). Stimulus type and design influence hemodynamic responses towards visual disgust and fear elicitors. Int J Psychophysiol.

[B12] Liu TT, Frank LR (2004). Efficiency, power, and entropy in event-related FMRI with multiple trial types. Part I: theory. Neuroimage.

[B13] Liu TT, Frank LR, Wong EC, Buxton RB (2001). Detection power, estimation efficiency, and predictability in event-related fMRI. Neuroimage.

[B14] Bandettini PA, Cox RW (2000). Event-related fMRI contrast when using constant interstimulus interval: theory and experiment. Magn Reson Med.

[B15] Janz C, Schmitt C, Speck O, Hennig J (2000). Comparison of the hemodynamic response to different visual stimuli in single-event and block stimulation fMRI experiments. J Magn Reson Imaging.

[B16] Le TH, Patel S, Roberts TP (2001). Functional MRI of human auditory cortex using block and event-related designs. Magn Reson Med.

[B17] Chee MW, Venkatraman V, Westphal C, Siong SC (2003). Comparison of block and event-related fMRI designs in evaluating the word-frequency effect. Hum Brain Mapp.

[B18] Spiering M, Everaerd W, Janssen E (2003). Priming the sexual system: implicit versus explicit activation. J Sex Res.

[B19] Spiering M, Everaerd W, Laan E (2004). Conscious processing of sexual information: mechanisms of appraisal. Arch Sex Behav.

[B20] Hoffmann H, Janssen E, Turner SL (2004). Classical conditioning of sexual arousal in women and men: effects of varying awareness and biological relevance of the conditioned stimulus. Arch Sex Behav.

[B21] van Lankveld JJ, Smulders FT (2008). The effect of visual sexual content on the event-related potential. Biol Psychol.

[B22] Oldfield RC (1971). The assessment and analysis of handedness: the Edinburgh inventory. Neuropsychologia.

[B23] Lang PJ, Bradley MM, Cuthbert BN (1999). The International Affective Picture System (IAPS).

[B24] Smolka MN, Buhler M, Klein S, Zimmermann U, Mann K, Heinz A, Braus DF (2006). Severity of nicotine dependence modulates cue-induced brain activity in regions involved in motor preparation and imagery. Psychopharmacology (Berl).

[B25] Klein S, Smolka MN, Wrase J, Grusser SM, Mann K, Braus DF, Heinz A (2003). The influence of gender and emotional valence of visual cues on FMRI activation in humans. Pharmacopsychiatry.

[B26] Smolka MN, Schumann G, Wrase J, Grüsser SM, Flor H, Mann K, Braus DF, Goldman D, Büchel C, Heinz A (2005). Catechol O-methyltransferase val158-met genotype affects processing of emotional stimuli in the amygdala and prefrontal cortex. J Neurosci.

[B27] Smolka MN, Buhler M, Schumann G, Klein S, Hu XZ, Moayer M, Zimmer A, Wrase J, Flor H, Mann K, Braus DF, Goldman D, Heinz A (2007). Gene-gene effects on central processing of aversive stimuli. Mol Psychiatry.

[B28] Bradley MM, Lang PJ (1994). Measuring emotion: the self-assessment manikin and the semantic differential. J Behav Ther Exp Psychiatry.

[B29] Kim SW, Sohn DW, Cho YH, Yang WS, Lee KU, Juh R, Ahn KJ, Chung YA, Han SI, Lee KH, Lee CU, Chae JH (2006). Brain activation by visual erotic stimuli in healthy middle aged males. Int J Impot Res.

[B30] Park K, Seo JJ, Kang HK, Ryu SB, Kim HJ, Jeong GW (2001). A new potential of blood oxygenation level dependent (BOLD) functional MRI for evaluating cerebral centers of penile erection. Int J Impot Res.

[B31] Phan KL, Wager T, Taylor SF, Liberzon I (2002). Functional neuroanatomy of emotion: a meta-analysis of emotion activation studies in PET and fMRI. Neuroimage.

[B32] Bradley MM, Sabatinelli D, Lang PJ, Fitzsimmons JR, King W, Desai P (2003). Activation of the visual cortex in motivated attention. Behav Neurosci.

[B33] Beauregard M, Levesque J, Bourgouin P (2001). Neural correlates of conscious self-regulation of emotion. J Neurosci.

[B34] Cunnington R, Windischberger C, Robinson S, Moser E (2006). The selection of intended actions and the observation of others' actions: a time-resolved fMRI study. Neuroimage.

[B35] Roesch MR, Olson CR (2007). Neuronal activity related to anticipated reward in frontal cortex: does it represent value or reflect motivation?. Ann N Y Acad Sci.

[B36] Shipp S (2004). The brain circuitry of attention. Trends Cogn Sci.

[B37] Tracy J, Goyal N, Flanders A, Weening R, Laskas J, Natale P, Waldron B (2007). Functional magnetic resonance imaging analysis of attention to one's heartbeat. Psychosom Med.

[B38] Kelley TA, Serences JT, Giesbrecht B, Yantis S (2008). Cortical mechanisms for shifting and holding visuospatial attention. Cereb Cortex.

[B39] Arnow BA, Desmond JE, Banner LL, Glover GH, Solomon A, Polan ML, Lue TF, Atlas SW (2002). Brain activation and sexual arousal in healthy, heterosexual males. Brain.

[B40] Ortigue S, Grafton ST, Bianchi-Demicheli F (2007). Correlation between insula activation and self-reported quality of orgasm in women. Neuroimage.

[B41] Mouras H, Stoleru S, Bittoun J, Glutron D, Pelegrini-Issac M, Paradis AL, Burnod Y (2003). Brain processing of visual sexual stimuli in healthy men: a functional magnetic resonance imaging study. Neuroimage.

[B42] Walter M, Bermpohl F, Mouras H, Schiltz K, Tempelmann C, Rotte M, Heinze HJ, Bogerts B, Northoff G (2008). Distinguishing specific sexual and general emotional effects in fMRI-subcortical and cortical arousal during erotic picture viewing. Neuroimage.

[B43] Oliveri M, Babiloni C, Filippi MM, Caltagirone C, Babiloni F, Cicinelli P, Traversa R, Palmieri MG, Rossini PM (2003). Influence of the supplementary motor area on primary motor cortex excitability during movements triggered by neutral or emotionally unpleasant visual cues. Exp Brain Res.

[B44] Moulier V, Mouras H, Pelegrini-Issac M, Glutron D, Rouxel R, Grandjean B, Bittoun J, Stoleru S (2006). Neuroanatomical correlates of penile erection evoked by photographic stimuli in human males. Neuroimage.

[B45] Mesulam MM, Mesulam MM and Davis FA (2006). Patterns in behavioral neuroanatomy: association areas, the limbic system, and behavioral specialization. Principles of Behavioral Neurology.

[B46] Decety J (1996). Do imagined and executed actions share the same neural substrate?. Brain Res Cogn Brain Res.

[B47] Stephan KM, Fink GR, Passingham RE, Silbersweig D, Ceballos-Baumann AO, Frith CD, Frackowiak RS (1995). Functional anatomy of the mental representation of upper extremity movements in healthy subjects. J Neurophysiol.

[B48] Amedi A, Malach R, Pascual-Leone A (2005). Negative BOLD differentiates visual imagery and perception. Neuron.

[B49] Janssen E, Carpenter D, Graham CA (2003). Selecting films for sex research: gender differences in erotic film preference. Arch Sex Behav.

